# Urinary Retention Following Methamphetamine and Cannabis Abuse in a 33-Year-Old Male

**DOI:** 10.7759/cureus.57033

**Published:** 2024-03-27

**Authors:** Noorvir Kaur, Maaz Haleem, Eduardo D Espiridion

**Affiliations:** 1 Psychiatry, Drexel University College of Medicine, West Reading, USA; 2 Psychiatry, Tower Health, West Reading, USA; 3 Psychiatry, Drexel University College of Medicine, Philadelphia, USA

**Keywords:** substance abuse, delirium, cannabis, methamphetamine, urinary retention

## Abstract

Urinary retention is described as an inability to voluntarily empty the bladder, with potential etiologies including mechanical obstruction and neurologic dysfunction. Abused substances like methamphetamine and cannabis can induce this dysfunction. We report a case about a patient with no prior psychiatric history with concomitant methamphetamine and cannabis use, presenting with an acute delirious state and urinary retention. Due to the multifactorial nature and acuity of a patient's presentation, clinicians should be aware of the potential for substance abuse to impact bladder function and consider this in patients who present with urinary symptoms, including urinary retention.

## Introduction

Methamphetamine is a highly addictive stimulant that affects the central nervous system (CNS). It is known to have adverse effects on the body, including cardiovascular complications, neurotoxicity, and psychiatric symptoms [1]. Methamphetamine primarily exerts its effects by inhibiting type 2 vesicular monoamine transporter (VMAT2) and the transmembrane dopamine transporter (DAT), leading to a surplus of dopamine released directly into the mesolimbic, mesocortical, and nigrostriatal pathways [2]. This excess dopamine release activates the brain’s reward system and creates a sense of euphoria, enhanced mental acuity, and a positive mood, which may lead to substance addiction.

Methamphetamine’s effects and toxicity on the kidneys have been studied and were found to be associated with acute kidney injury [2], though these were observed to be mild following immediate resolution. The connection between urinary retention and methamphetamine use has not been studied. The micturition reflex itself involves a complex interaction of detrusor muscle contraction accompanied by relaxation of the urinary sphincter [3]. Urinary retention may come about due to disruption of these physiologic pathways, as seen with anticholinergic agents that inhibit detrusor contraction. It is proposed that methamphetamines exert an alpha-agonist effect indirectly by increasing the release of endogenous norepinephrine [4]. Because the bladder neck is innervated primarily by alpha-adrenergic receptors, excessive and prolonged stimulation of these receptors by norepinephrine may lead to urinary retention [5]. 

Cannabis use has also been associated with various urinary symptoms, including urinary retention [6]. Cannabinoids, the active components within cannabis, produce a wide spectrum of central and peripheral effects. The functional role of cannabinoid receptors in the urinary bladder has been studied, with findings suggesting that cannabis can impact urinary bladder function [7]. It has been demonstrated that cannabinoids act principally as prejunctional modulators of neurotransmission. This can indirectly affect the micturition process by affecting the nociceptive response pathways. It is likely that the cannabinoid receptors located in the periphery, such as in the bladder, participate in the intrinsic control of the initiation of afferent stimulus, possibly impairing it [7].

## Case presentation

A 33-year-old male was brought to the emergency room of a local hospital after he was found unresponsive at his workplace. His employer expressed concerns that he may have consumed a substance during his lunch break. The Emergency Medical Service (EMS) administered naloxone in the field with no improvement. The patient was not cyanotic. He reportedly woke up a few minutes later and became very agitated and combative. Midazolam was administered to control his behavior. After the patient had calmed down, he reported to hospital staff that he snorted, smoked, and consumed methamphetamines. In addition, the patient had consumed some marijuana edibles. During this interview, hospital staff noticed that he became progressively lethargic and was allowed to sleep. When the patient was revisited after an hour, he was observed to be hypervigilant. He complained of urinary retention, but he refused any attempts at straight catheterization. He then became violent, so he was put under physical restraints, and Olanzapine 10 mg intramuscularly was administered. Table 1 shows the relevant laboratory findings upon admission. A liter of urine was obtained after straight catheterization. However, the patient continued to have lower abdominal pain and urinary retention, so another straight catheter was inserted, and another half liter of urine was removed. He was subsequently admitted to the medical floor. The patient was not taking any prescription medication prior to this hospitalization.

**Table 1:** Relevant laboratory findings BUN: blood urea nitrogen, GFR: glomerular filtration rate, THC: tetrahydrocannabinol

Laboratory test	Results	Reference Values
Serum Na+	139 mmol/L	136–145 mmol/L
Serum K+	4.4 mmol/L	3.5–5.1 mmol/L
Serum creatinine	0.95 mg/dL	0.73–1.18 mg/dL
BUN	10 mg/dL	9–23 mg/dL
Estimated GFR chronic kidney disease epidemiology collaboration eGFR (CKD-EPI)	107 mL/min/1.73m*2	>60 mL/min/1.73 m^2^
Glucose	113 mg/dL	74–99 mg/dL
Hemoglobin	15.7 g/dL	14.0–17.5 g/dL
Hematocrit	45.6 %	39.0–53.0%
White blood cell count	12.3 x 10^9^/L	4.8–10.8 x 10^9^/L
Amphetamine screen (urine)	Positive	Negative
Opioid screen (urine)	Negative	Negative
Benzodiazepine screen (urine)	Positive	Negative
THC (marijuana urine) screen	Positive	Negative

**Figure 1:**
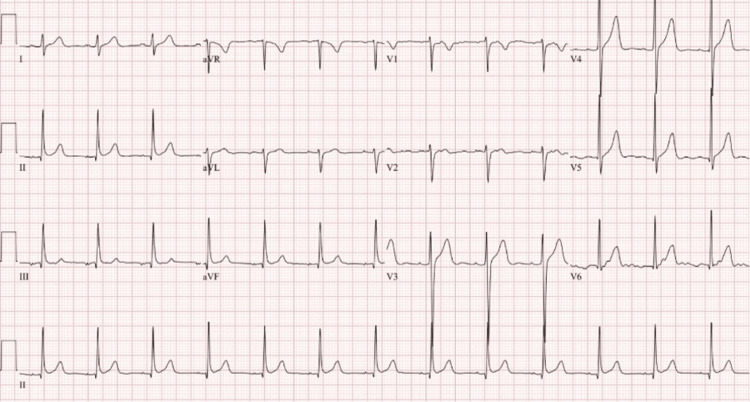
EKG on admission showing no signs of ischemia or arrhythmias

**Figure 2:**
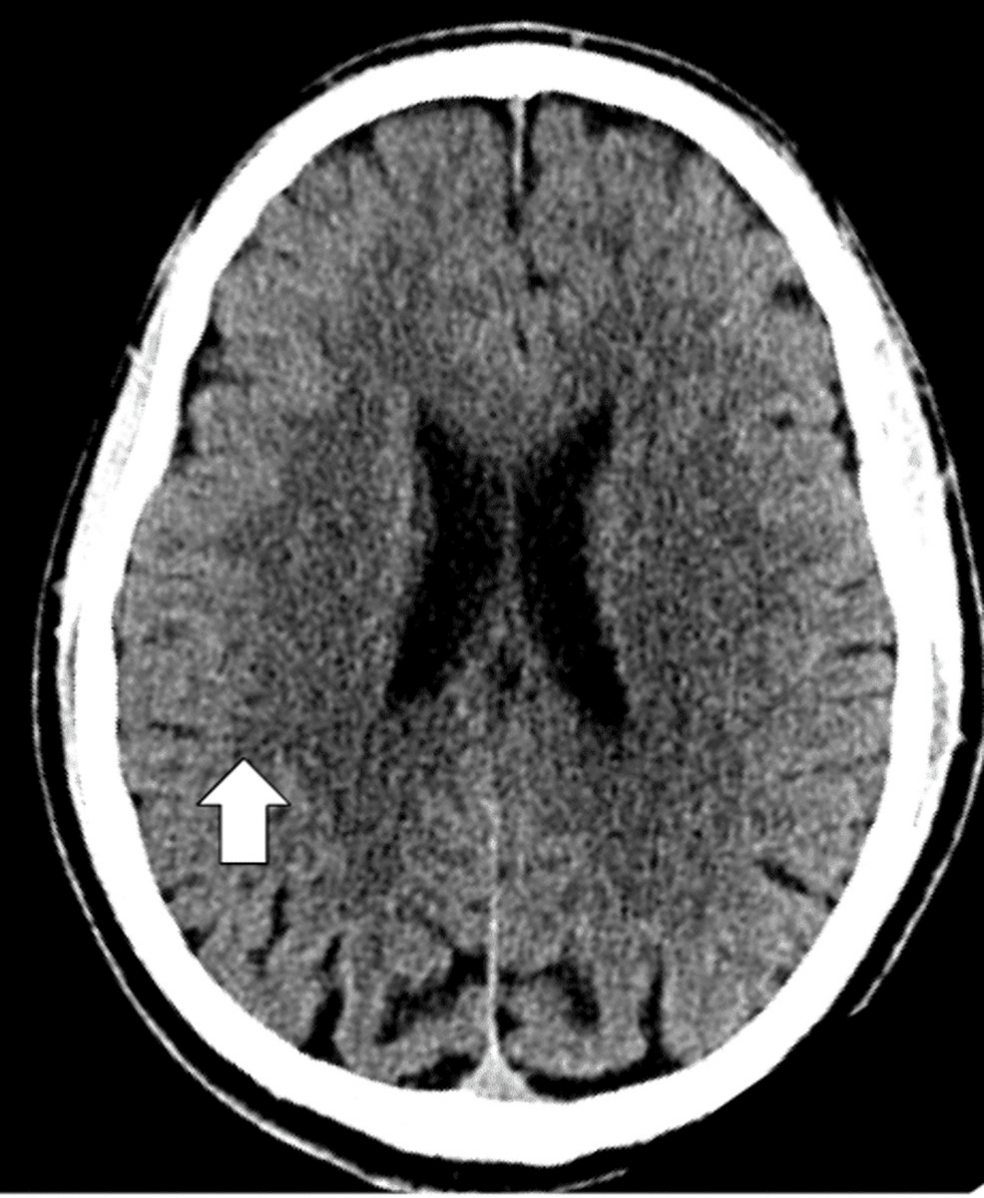
CT head or brain without contrast showing no signs of an acute infarct or hemorrhage with normal grey-white differentiation depicted by white arrow

In addition, an EKG and CT scan of the head were done to rule out any organic causes for the patient's presentation. The EKG is shown in Figure 1, normal sinus rhythm with a ventricular rate of 79 beats per minute with a QTc of 410 milliseconds, and no signs of arrhythmias or myocardial infarctions. The CT of the head without contrast demonstrated in Figure 2 shows no signs of an acute infarct or intracranial hemorrhage, with mild cerebral atrophy.

The following day, the patient still had dysuria and urinary hesitancy but could void urine on his own. However, he noted referential and paranoid ideations that the staff were talking about him. He had déjà vu feelings as if he were at a local car shop and noted auditory hallucinations of voices commenting on his behaviors. He stated that he had experienced this before whenever he used methamphetamines. He did receive Olanzapine 5 mg orally on two occasions. On the third day, the patient did not experience any urinary or psychotic symptoms. After declining inpatient substance abuse treatment referrals, he was discharged from the hospital thereafter. The patient then went on to self-register for an addiction drug rehabilitation program. 

## Discussion

Methamphetamine is a sympathomimetic amine of the central nervous system, and it produces potent CNS-mediated stimulant, anorectic, and cardiovascular effects that have been used in the treatment of psychiatric disorders such as narcolepsy and ADHD. Consumption of methamphetamine has also been reported to cause neurogenic bladder and urinary retention [8]. This study reports the effects of methamphetamine on the urinary system that led to significant urological complications. Management of methamphetamine-induced urinary retention involves addressing the acute urinary retention initially, which may require catheterization to relieve the bladder [9]. Methamphetamines have adverse effects that are associated with the onset of delusions and hallucinations in patients with or without any pre-existing psychotic disorders [10]. In one such case report, a patient diagnosed with ADHD one month prior experienced a first episode of substance-induced psychosis relating to the overuse of amphetamines [11]. As can be seen within this patient, he experienced hallucinations, delusions, and disorganized thinking, comprising a substance-induced psychotic episode. This is not uncommon, as recent studies have also explored the relationship between methamphetamine use and psychosis. One study reported that methamphetamine use is associated with an increased risk of psychotic symptoms in the general population and that this increased risk is confined to people who use it at least weekly [12].

Methamphetamine use has been associated with urinary retention, a condition characterized by impaired emptying of the bladder resulting in post-void residual urine. This effect is thought to occur due to methamphetamine's indirect alpha-agonist effect, which increases the release of endogenous norepinephrine stores. The alpha-adrenergic stimulation can lead to increased bladder neck and urethral sphincter tone, thereby causing urinary retention [1]. The dopaminergic VMAT2 and DAT pathways that methamphetamine acts on exert an inhibitory effect on voiding by acting through the alpha 1 receptor on the internal urethral sphincter. Hence, urinary retention occurs when there is a loss of coordination of the detrusor muscle contraction with the relaxation of the urinary sphincter.

A review study further supports the therapeutic benefits of alpha-blockers and anticholinergics in methamphetamine abusers with lower urinary tract symptoms [13]. 

The relationship between marijuana use and urinary retention is complex and not as well-documented as with other substances. However, there are reports and studies that suggest marijuana can impact bladder function in various ways. For instance, a study published in the Journal of the American Medical Association (JAMA) in 1979 reported urinary retention following cannabis ingestion, highlighting that this condition can occur in the context of marijuana use [6]. This suggests that, like other substances, marijuana might affect the urinary system, potentially leading to urinary retention. The mechanisms by which marijuana might cause urinary retention are not fully understood but could involve the effects of cannabinoids on the central nervous system and their interaction with cannabinoid receptors in the bladder [7]. Some studies have found that cannabinoids can lead to a significant reduction in urge incontinence episodes and improvement in bladder control, indicating a potential therapeutic role for cannabinoids in managing overactive bladder symptoms [7]. However, it is important to note that the effects of marijuana on the bladder and urinary retention can vary widely among individuals, and more research is needed to fully understand these effects and their clinical implications.

## Conclusions

Acute urinary retention is a medical condition that requires immediate medical attention. Methamphetamine and cannabis abuse are associated with an increased risk of urinary retention. Although this is not a commonly seen adverse effect of methamphetamine and cannabis abuse, urinary retention may occur. Healthcare providers should be aware of this potential complication among individuals with substance use disorders, and they should consider screening for urinary symptoms in these patient populations.
